# Impact of hydration status on haemodynamics, effects of acute blood pressure‐lowering treatment, and prognosis after stroke

**DOI:** 10.1111/bcp.13761

**Published:** 2018-10-10

**Authors:** Charlotte K. Billington, Jason P. Appleton, Eivind Berge, Nikola Sprigg, Mark Glover, Philip M. W. Bath

**Affiliations:** ^1^ Division of Respiratory Medicine University of Nottingham Nottingham UK; ^2^ Stroke Trials Unit, Division of Clinical Neuroscience University of Nottingham Nottingham UK; ^3^ Department of Internal Medicine and Cardiology Oslo University Hospital Oslo Norway

**Keywords:** glyceryl trinitrate, acute stroke, blood pressure, dehydration, intracerebral haemorrhage, ischaemic stroke

## Abstract

**Aims:**

Although high blood pressure (BP) is common in acute stroke and associated with poor outcome, the Efficacy of Nitric Oxide in Stroke (ENOS) trial showed no beneficial effect of antihypertensive treatment in this situation. Antihypertensive agents have accentuated effects in dehydrated patients. We assessed the impact of dehydration on haemodynamics, the effects of antihypertensive treatment, and prognosis in the ENOS trial.

**Methods:**

ENOS randomized 4011 patients with acute stroke and raised systolic BP to a glyceryl trinitrate (GTN) patch or no GTN patch, and to continue or to stop existing antihypertensive treatment within 48 h of onset. The primary outcome was functional outcome (modified Rankin Scale, mRS) at day 90. Blood markers of dehydration at baseline were collected at two sites (*n* = 310) and their relationship with haemodynamics and outcome was assessed.

**Results:**

There were no significant associations between dehydration markers and fall in blood pressure from baseline to day 1, and no significant interaction with allocated treatment. Overall, increasing urea was associated with an unfavourable shift in mRS [odds ratio 3.43, 95% confidence interval (CI) 1.42, 8.32; *P* = 0.006] and increased risk of death at day 90 (hazard ratio 4.55, 95% CI 1.51, 13.66; *P* = 0.007).

**Conclusions:**

Blood pressure‐lowering treatment was safe in dehydrated patients, with no precipitous changes in BP, thus supporting its use in acute stroke prior to blood markers of dehydration becoming available. Increased baseline urea was associated with poor prognosis after stroke.

## What is Already Known about this Subject

- Both high blood pressure (BP) and dehydration are common and independently associated with poor clinical outcomes following acute stroke.
- Antihypertensive agents may have accentuated effects in dehydrated patients, but their effects in dehydrated acute stroke patients are unclear.

## What this Study Adds

- BP‐lowering treatment was safe in dehydrated patients, with no precipitous changes in BP, thus supporting its use in acute stroke prior to blood markers of dehydration becoming available.

## Introduction

High blood pressure (BP) is common in acute stroke and associated independently with a poor outcome in both ischaemic stroke and intracerebral haemorrhage 1. Lowering elevated BP is recommended in acute intracerebral haemorrhage 2, and is safe in ischaemic stroke 3, 4. Most drug classes that might be useful for lowering BP (including angiotensin‐converting enzyme inhibitors, angiotensin receptor antagonists and nitrates) have accentuated vasodepressant effects when patients are dehydrated or hypovolaemic 5. Reduced circulating volume is also common in stroke, especially if admission to hospital is delayed, thereby allowing dehydration to develop. Hypovolaemia may reduce cerebral perfusion and increase the infarct core in ischaemic stroke 6 and the perihaematomal ischaemia in intracerebral haemorrhage 7. It may also lead to renal impairment and is associated with venous thromboembolism 8, 9. As a consequence, dehydration has been associated with poor clinical outcomes following acute stroke 10, 11, 12, and adequate hydration after stroke is recommended in clinical guidelines 8.

The Efficacy of Nitric Oxide in Stroke (ENOS) trial assessed the effect of transdermal glyceryl trinitrate (GTN) on outcome in 4011 patients 4. Overall, GTN did not alter clinical outcomes, despite lowering BP by 7/3.5 mmHg at day 1 4, but when administered within 6 h of stroke onset, GTN improved multiple clinical outcomes at day 90 13. The aim of the present planned substudy was to assess the impact of dehydration on haemodynamic changes, effect of blood pressure reduction on clinical outcomes, and prognosis after stroke.

## Methods

The ENOS trial protocol, statistical analysis plan, baseline characteristics and main trial results have been published elsewhere 4, 14, 15, 16. In brief, ENOS recruited 4011 people with acute stroke within 48 h of onset and high systolic BP (140–220 mmHg), and randomized them to a GTN 5 mg patch or no patch for 7 days. In addition, participants taking antihypertensive medication prior to the index event were randomized to continue or stop these drugs for 7 days. Patients or relatives/carers gave written consent to participate. ENOS was registered (ISRCTN99414122) and approved by ethics committees/competent authorities in all participating countries.

### Biomarkers of dehydration

Biochemical biomarkers were recorded at two sites (Nottingham City Hospital and Queen's Medical Centre, Nottingham, UK), including full blood count (haemoglobin, red cell count, haematocrit/packed cell volume) and biochemistry (sodium, potassium, urea, creatinine, glucose). Estimated glomerular filtration rate (eGFR) was calculated using the Modification of Diet in Renal Diseases (MDRD) equation: 186 × serum creatinine (μmol l^–1^)^–1.154^ × age (years)^–0.203^ × (0.742 if female) × (1.210 if Black). As ethnicity was not recorded in ENOS, participants of Black ethnicity will have an underestimated eGFR.

In addition, we calculated the following markers of dehydration: (i) urea : creatinine (mg dl^–1^); (ii) 2Na + glucose + urea (mmol l^–1^) 17;, and (iii) 2Na + 2K + glucose + urea (mmol l^–1^) 18, which are all elevated in dehydrated patients. The latter two are also formulae for osmolarity. Ethanol may be added to the calculations, to refine the estimate of osmolarity, but was not measured in ENOS. Furthermore, ENOS did not routinely collect information on clinical markers of dehydration, such as thirst or skin turgor.

Dehydration was defined as: Na > 145 mmol l^–1^, urea > 7.5 mmol l^–1^, urea : creatinine ratio >20, calculated osmolarity >297 mmol l^–1^, eGFR <30 ml min^–1^ 1.73m^–2^, haematocrit >0.54 l l^–1^ for men and >0.47 l l^–1^ for women, and red cell count >6.5 cells μl^–1^ for men and >5.8 cells μl^–1^ for women.

### Haemodynamic outcomes

Peripheral haemodynamics (BP and heart rate) were measured at baseline (three measurements) and on days 1 to 7 (two measurements per day), using a validated automated monitor (OMRON Healthcare Company, Kyoto, Japan) 19.

### Clinical outcomes

The primary outcome (functional outcome) was measured using the modified Rankin Scale (mRS, a seven‐level ordered categorical scale, where 0 = independent and 6 = dead) at day 90. Clinical outcomes included all‐cause death at day 90, and headache, hypotension, hypertension at day 7, and change in the Scandinavian Stroke Scale (SSS, a marker of neurological improvement) from baseline to day 7. Multiple secondary outcomes were also assessed at day 90. All day 90 outcomes were assessed centrally by telephone by trained investigators based at the International Coordinating Centre in Nottingham; assessors were masked to treatment allocation.

### Statistical analysis

Data were analysed by intention‐to‐treat, in line with the ENOS trial statistical analysis plan and statistical analyses adopted in the primary publication 16. Data are shown as number (%), median [interquartile range], or mean (standard deviation). Baseline characteristics between groups were assessed using the chi‐squared test for categorical variables, and one‐way analysis of variance for continuous variables.

Associations between dehydration markers and haemodynamic changes from baseline to day 1 were assessed using multiple linear regression, after adjustment for age, sex and allocated treatment, with resultant standardized regression coefficients (β) given. Interaction *P*‐values were calculated by adding an interaction term for treatment and dehydration marker to the models.

Associations between dehydration markers and clinical outcomes were assessed using Cox proportional hazard regression, binary logistic regression, multiple linear regression or ordinal logistic regression. The impact of dehydration on the effect of treatment was assessed by introducing an interaction term for treatment and dehydration status to the analyses. Statistical models were adjusted for prognostic baseline covariates, including age, sex, systolic BP, SSS and time to randomization. Analyses involving the whole population were also adjusted for treatment allocation. The resultant hazard ratio (HR), mean difference (MD) or odds ratio and associated 95% confidence intervals are given, with significance set at *P* ≤ 0.05. Analyses were performed using SPSS version 22 (SPSS Inc., Chicago, IL, USA).

## Results

The present sub‐study included 310 participants (GTN 158, no GTN 152; continue prestroke antihypertensive agents 74, temporarily stop 76) from two trial sites in Nottingham, UK. Of these, 294 had data on one or more laboratory measure of dehydration. Baseline characteristics and biochemical markers of dehydration are shown in Table 1. Clinical characteristics were well balanced, and blood markers of dehydration did not differ between randomized groups (GTN *vs*. no GTN, or continue *vs*. stop prestroke antihypertensive agents), except for raised haematocrit (Table 1).

**Table 1:** Baseline characteristics of patients enrolled into the Efficacy of Nitric Oxide in Stroke (ENOS) sub‐study. Data are are given as number (%) or mean (standard deviation); comparison by the chi‐square test or one‐way analysis of variance

Participants	All										
	310	GTN	*n*	No GTN	*n*	*P*	Continue	*n*	Stop	*n*	*P*
**Age (years)**	73.2 (11.7)	73.1 (11.7)	158	73.3 (11.7)	152	–	77.0 (9.2)	74	76.1 (8.9)	76	–
**Sex, male (%)**	169 (54.5)	84 (53.2)	158	85 (55.9)	152	–	36 (48.6)	74	33 (43.4)	76	–
**Drugs prestroke (%)**											
**ACE‐I**	57 (18.4)	26 (16.5)	158	31 (20.4)	152	–	30 (40.5)	74	26 (34.2)	76	–
**ARB**	18 (5.8)	12 (7.6)	158	6 (3.9)	152	–	7 (9.5)	74	10 (13.2)	76	–
**β‐receptor antagonist**	68 (21.9)	39 (24.7)	158	29 (19.1)	152	–	28 (37.8)	74	36 (47.4)	76	–
**Calcium channel blocker**	56 (18.1)	34 (21.5)	158	22 (14.5)	152	–	25 (33.8)	74	25 (32.9)	76	–
**Diuretic**	62 (20)	38 (24.1)	158	24 (15.8)	152	–	24 (32.4)	74	31 (40.8)	76	–
**Others**	14 (4.5)	11 (7)	158	3 (2)	152	–	8 (10.8)	74	5 (6.6)	76	–
**Stroke type, ischaemic (%)**	264 (85.2)	131 (82.9)	158	133 (87.5)	152	–	66 (89.2)	74	66 (86.8)	76	–
**Stroke severity (SSS, /58)**	32.5 (13.9)	32.4 (13.9)	158	32.6 (14)	152	–	33.0 (13.7)	74	32.2 (13.1)	76	–
**Stroke syndrome, TACS (%)**	124 (40)	70 (44.3)	158	54 (35.5)	152	–	33 (44.6)	74	30 (39.5)	76	–
**Systolic blood pressure (mmHg)**	166.6 (20.1)	167.7 (19.1)	158	165.4 (21.1)	152	–	166.9 (19.9)	74	165.0 (18.0)	76	–
**Diastolic blood pressure (mmHg)**	89.1 (14.3)	89.7 (15.5)	158	88.4 (12.9)	152	–	87.1 (14.3)	74	85.2 (12.6)	76	–
**Heart rate (bpm)**	75.4 (14.4)	74.9 (13.8)	158	76 (14.9)	152	–	77.3 (16.4)	74	73.6 (14.1)	76	–
**Time, onset to randomization (h)**	27.6 (12.1)	27.5 (12.2)	158	27.8 (12.0)	152	–	25.4 (11.7)	74	25.6 (12.0)	76	–
**Blood analyses**											
**Haematocrit (l l** ^**–1**^ **)**	0.42 (0.04)	0.42 (0.04)	152	0.41 (0.04)	142	0.33	0.41 (0.04)	69	0.41 (0.04)	70	0.61
**Haemoglobin (g l** ^**–1**^ **)**	139 (16.3)	140 (16.5)	152	138 (16.1)	142	0.30	136 (14.2)	69	139 (15.0)	70	0.31
**Red cell count (10^12^ l** ^**–1**^ **)**	4.6 (0.5)	4.6 (0.5)	152	4.6 (0.5)	142	0.54	4.5 (0.5)	69	4.6 (0.5)	70	0.56
**Sodium (mmol l** ^**–1**^ **)**	138.3 (3.6)	138.3 (3.9)	154	138.4 (3.4)	143	0.69	138.1 (4.0)	70	138.5 (3.8)	72	0.57
**Potassium (mmol l** ^**–1**^ **)**	4.1 (0.5)	4.1 (0.5)	149	4.2 (0.4)	141	0.16	4.0 (0.5)	67	4.0 (0.5)	71	0.92
**Urea (mmol l** ^**–1**^ **)**	6.5 (2.6)	6.4 (2.6)	154	6.6 (2.6)	143	0.45	7.1 (2.7)	70	7.0 (2.2)	72	0.87
**Creatinine (μmol l** ^**–1**^ **)**	94.4 (32)	93.1 (25.3)	154	95.8 (37.9)	143	0.47	98.6 (43.4)	70	97.7 (25.4)	72	0.88
**eGFR (ml min** ^**–1**^ **1.73** **m** ^**2**^ **)**	69.7 (20.8)	69.5 (19.7)	154	69.9 (22.1)	143	0.85	65.4 (19.3)	70	63.2 (18.3)	72	0.51
**Glucose (mmol l** ^**–1**^ **)**	6.7 (1.9)	6.7 (1.9)	126	6.7 (1.9)	114	0.93	6.8 (1.9)	59	6.9 (1.7)	52	0.87
**Urea : creatinine (mg dl** ^**–1**^ **)**	17.4 (5.3)	17.0 (4.6)	154	17.8 (5.9)	143	0.21	18.4 (4.9)	70	18.0 (4.6)	72	0.67
**Osmolarity A (mmol l** ^**–1**^ **)**	289.6 (7.7)	288.9 (8.5)	126	290.3 (6.7)	112	0.17	289.0 (8.9)	59	291.5 (7.7)	51	0.13
**Osmolarity B (mmol l** ^**–1**^ **)**	297.8 (7.8)	297.2 (8.7)	122	298.6 (6.7)	111	0.17	296.8 (9.3)	56	299.3 (7.8)	50	0.14
**Dehydration markers**											
**Sodium >145 mmol l** ^**–1**^ **(%)**	3 (1)	3 (1.9)	154	0 (0)	143	0.09	1 (1.4)	70	1 (1.4)	72	0.98
**Urea >7.5 mmol l** ^**–1**^ **(%)**	69 (23.2)	35 (22.7)	155	34 (23.8)	143	0.83	20 (28.6)	70	25 (34.7)	72	0.43
**eGFR <30 ml min** ^**–1**^ **1.73** **m** ^**2**^ **(%)**	4 (1.3)	1 (0.6)	154	3 (2.1)	143	0.28	2 (2.9)	70	0 (0)	72	0.15
**Urea : creatinine >20 (%)**	75 (25.3)	36 (23.4)	154	39 (27.3)	143	0.44	26 (37.1)	70	19 (26.4)	72	0.17
**Osmolarity A >297 mmol l** ^**–1**^ **(%)**	31 (13)	17 (13.5)	126	14 (12.5)	112	0.82	10 (16.9)	59	13 (25.5)	51	0.27
**Osmolarity B >297 mmol l** ^**–1**^ **(%)**	129 (55.6)	62 (51.2)	121	67 (60.4)	111	0.16	30 (53.6)	56	31 (62.0)	50	0.38
**Haematocrit >0.54 l l** ^**–1**^ **(male), >0.47 l l** ^**–1**^ **(female) (%)**	6 (2.0)	6 (3.9)	152	0 (0)	142	0.017	1 (1.4)	69	1 (1.4)	70	0.99
**Haemoglobin >180 g l** ^**–1**^ **(male), >165 g l** ^**–1**^ **(female) (%)**	3 (1)	3 (2)	152	0 (0)	142	0.09	1 (1.4)	69	1 (1.4)	70	0.99
**Red cell count >6.5 (male), >5.8 (female) (%)**	0 (0)	0 (0)	152	0 (0)	142	–	0 (0)	69	0 (0)	70	–

Osmolarity A: 2Na + Glucose + Urea; Osmolarity B: 2Na + 2K + Glucose+ Urea (mmol l^–1^). ACE‐I, angiotensin‐converting enzyme inhibitor; ARB, angiotensin receptor blocker; bpm, beats per minute; eGFR, estimated glomerular filtration rate; GTN, glyceryl trinitrate; TACS, total anterior circulation syndrome; SSS, Scandinavian Stroke Scale

The relationship between blood biomarkers of dehydration and change in haemodynamic parameters from baseline to day 1 is shown split by randomization to GTN or no GTN, and stop or continue prestroke antihypertensive agents (Table 2). There were no significant interactions between treatment with GTN *vs*. no GTN and dehydration markers on the change in BP and heart rate from baseline to day 1. GTN lowered BP and increased heart rate, but dehydration markers did not significantly influence these findings. Similarly, there were no significant interactions between continuing *vs*. stopping prestroke antihypertensive agents and dehydration markers on haemodynamic changes from baseline to day 1.

**Table 2:** Relationship between blood measures of hydration status and allocated treatment and percentage change in systolic blood pressure, diastolic blood pressure and heart rate from day 0 to day 1, in patients randomized to glyceryl trinitrate (GTN; *n* = 152) and no GTN (*n* = 148), and patients randomized to continue (*n* = 74) and stop (*n* = 76) prestroke antihypertensive agents. Multiple linear regression with adjustment for age and sex, allocated treatment and hydration status. Data are given as standardized regression coefficient (β) with associated *P*‐values

**Allocated treatment (Rx)**	**MHS**	**ΔSBP**					**ΔDBP**					**ΔHR**				
		**β for Rx**	***P***	**β for MHS**	***P***	***P***_**interaction**_	**β for Rx**	***P***	**β for MHS**	***P***	***P***_**interaction**_	**β for Rx**	***P***	**β for MHS**	***P***	***P***_**interaction**_
**GTN/No GTN**	Sodium	−0.264	<0.001	0.175	0.35	0.64	−0.143	0.015	0.014	0.81	0.54	0.158	0.008	−0.029	0.62	0.08
	Urea	−0.263	<0.001	0.019	0.76	0.50	−0.135	0.021	0.138	0.027	0.34	0.156	0.009	−0.032	0.61	0.64
	eGFR	−0.264	<0.001	−0.002	0.97	0.57	−0.143	0.015	−0.045	0.49	0.67	0.158	0.008	0.054	0.41	0.24
	Urea : creatinine	−0.261	<0.001	0.045	0.47	0.37	−0.135	0.021	0.096	0.13	0.54	0.161	0.007	0.036	0.58	0.54
	Osmolarity A	−0.257	<0.001	0.007	0.92	0.54	−0.198	0.003	−0.085	0.19	0.73	0.190	0.004	−0.082	0.21	0.12
	Osmolarity B	−0.256	<0.001	0.007	0.91	0.59	−0.205	0.002	−0.073	0.26	0.71	0.201	0.003	−0.084	0.21	0.15
	Haematocrit	−0.258	<0.001	0.003	0.96	0.34	−0.134	0.024	−0.039	0.54	0.26	0.158	0.008	0.011	0.86	0.83
	Haemoglobin	−0.257	<0.001	−0.007	0.91	0.45	−0.132	0.026	−0.057	0.37	0.23	0.156	0.009	0.028	0.66	0.89
	Red cell count	−0.259	<0.001	0.045	0.46	0.78	−0.134	0.023	−0.053	0.39	0.08	0.159	0.007	−0.021	0.74	0.36
**Continue/stop**	Sodium	−0.112	0.19	0.013	0.88	0.51	0.024	0.78	−0.027	0.76	0.98	−0.043	0.62	−0.080	0.35	0.88
	Urea	−0.112	0.20	−0.038	0.67	0.80	0.024	0.78	0.050	0.57	0.27	−0.042	0.63	0.041	0.64	0.98
	eGFR	−0.111	0.20	−0.021	0.82	0.89	0.025	0.77	−0.001	0.99	0.59	−0.044	0.61	0.039	0.66	0.85
	Urea : creatinine	−0.114	0.19	0.024	0.80	0.35	0.018	0.83	0.108	0.24	0.90	−0.045	0.60	0.065	0.47	0.68
	Osmolarity A	−0.111	0.27	−0.029	0.77	0.74	−0.061	0.54	−0.154	0.12	0.84	−0.075	0.45	−0.131	0.18	0.63
	Osmolarity B	−0.100	0.33	−0.036	0.72	0.62	−0.040	0.70	−0.137	0.17	0.63	−0.062	0.54	−0.129	0.20	0.71
	Haematocrit	−0.097	0.27	−0.063	0.48	0.83	0.036	0.68	−0.069	0.44	0.14	−0.040	0.65	−0.057	0.52	0.16
	Haemoglobin	−0.102	0.25	−0.081	0.37	0.57	0.030	0.73	−0.102	0.26	0.09	−0.041	0.64	−0.040	0.66	0.28
	Red cell count	−0.095	0.28	0.010	0.12	0.56	0.034	0.70	−0.085	0.35	0.70	−0.040	0.65	−0.027	0.76	0.14

β, standardized regression coefficient; interaction *P*, allocated treatment*blood measure of hydration status; DBP, diastolic blood pressure; eGFR, estimated glomerular filtration rate; MHS, measure of hydration status; Rx, allocated treatment; SBP, systolic blood pressure

We compared the effects of BP‐lowering treatment (GTN *vs*. no‐GTN, and continue *vs*. stop) on neurological impairment and clinical events during the first 7 days and outcome at 3 months, by level of urea (Table 3) and level of urea : creatinine ratio (Table S1). There were no differences in change in neurological impairment or in rates of reported hypotension, hypertension or headache by day 7 in those randomized to GTN compared with no GTN, or those randomized to stop *vs*. continue their prestroke antihypertensive agents. In those with a raised urea (>7.5 mmol l^–1^), there was a tendency towards an unfavourable shift in mRS and increased death at 90 days when randomized to GTN *vs*. no GTN (*P* for interaction = 0.047 and 0.050, respectively), a finding not seen with a raised urea : creatinine ratio. No significant interactions were noted in regard to stop *vs*. continue.

**Table 3:** Effects of GTN *vs*. non‐GTN, and effects of continue *vs*. stop antihypertensive treatment on change in neurological status and clinical events during the first 7 days and outcome at 3 months, by hydration status, given by level of urea. Multiple linear regression, binary logistic regression, ordinal logistic regression or Cox proportional hazards regression with adjustment for age, sex and time to randomization. Data are given as *n* (%), mean (standard deviation), mean difference (MD), odds ratio (OR) or hazard ratio (HR) with 95 confidence intervals (CIs)

	**Urea**	**All**	**GTN**	**No GTN**	**OR/MD/HR (95% CI)**	***P***	***P***_**interaction**_	**Continue**	**Stop**	**OR/MD/HR (95% CI)**	***P***	***P***_**interaction**_
**Day 7**		*n* = 310	*n* = 158	*n* = 152				*n* = 74	*n* = 76			
**ΔSSS 0–7**	>7.5	2.1 (10.5)	0.7 (12.6)	3.6 (7.7)	−3.02 (−8.33, 2.29)	0.26	0.25	−1.1 (10.3)	3.0 (12.8)	−4.98 (−12.59, 2.63)	0.19	0.16
	<7.5	5.6 (10.0)	5.8 (10.0)	5.5 (10.1)	0.06 (−2.63, 2.75)	0.97	–	4.9 (11.1)	3.5 (10.3)	1.85 (−2.70, 6.39)	0.42	–
**Hypotension (%)**	>7.5	4 (5.8)	4 (11.4)	0 (0)	–	–	–	3 (15.0)	1 (4.0)	8.59 (0.42, 177.24)	0.16	0.07
	<7.5	5 (2.2)	4 (3.4)	1 (0.9)	3.77 (0.41, 34.84)	0.24	–	1 (2.0)	3 (6.4)	0.99 (0.87, 1.13)	0.86	–
**Hypertension (%)**	>7.5	3 (4.3)	1 (2.9)	2 (5.9)	0.31 (0.02, 4.47)	0.39	0.31	0 (0)	2 (8.0)	–	–	–
	<7.5	10 (4.4)	7 (5.9)	3 (2.8)	2.42 (0.57, 10.29)	0.23	–	2 (4.0)	1 (2.1)	2.32 (0.15, 34.96)	0.54	–
**Headache (%)**	>7.5	11 (15.9)	6 (17.1)	5 (14.7)	1.28 (0.32, 5.11)	0.72	0.31	4 (20.0)	2 (8.0)	2.24 (0.34, 14.70)	0.40	0.22
	<7.5	41 (18.0)	29 (24.4)	12 (11.0)	2.76 (1.30, 5.87)	0.008	–	3 (6.0)	5 (10.6)	0.65 (0.14, 3.02)	0.58	–
**Day 90**												
**Functional outcome (mRS)** a	>7.5	3.5 (1.9)	3.9 (1.9)	3.1 (1.7)	2.19 (0.92, 5.26)	0.08	0.047	3.2 (1.8)	3.6 (1.9)	0.57 (0.19, 1.67)	0.30	0.26
	<7.5	3.0 (1.6)	3.0 (1.6)	3.0 (1.6)	0.96 (0.61, 1.52)	0.87	–	3.4 (1.7)	3.3 (1.5)	1.14 (0.56, 2.34)	0.72	–
**Death (%)**	>7.5	15 (21.7)	11 (31.4)	4 (11.8)	4.42 (1.26, 15.44)	0.020	0.050	3 (15.0)	6 (24.0)	0.53 (0.12, 2.31)	0.40	0.51
	<7.5	19 (8.4)	10 (8.5)	9 (8.3)	1.08 (0.43, 2.75)	0.87	–	6 (12.0)	5 (10.6)	1.47 (0.37, 5.87)	0.59	–

BP, blood pressure; CI, confidence interval; GTN, glyceryl trinitrate; mRS, modified Rankin Scale; OR, odds ratio; SD, standard deviation; SSS, Scandinavian Stroke Scale

a Ordinal logistic regression

The associations between markers of dehydration and mRS and death at day 90 were assessed across the total available population (Table 4). High urea at baseline was associated with an unfavourable shift in mRS at day 90 and increased death at day 90. Although there was no significant association between baseline creatinine and mRS at day 90, increasing creatinine was associated with an increased risk of death at day 90. By contrast, a high sodium at baseline was associated with a favourable shift in mRS at day 90. We did not find any significant relationships in adjusted analyses between other markers of dehydration and clinical outcomes at day 90 (Table S2).

**Table 4:** Relationships between baseline markers of dehydration and modified Rankin Scale (mRS) or death at 3 months. Analysis by ordinal logistic regression or Cox proportional hazards regression; with adjustment for age, sex, systolic blood pressure, stroke severity (Scandinavian Stroke Scale), time from onset to randomization, continue/stop and GTN/no GTN. Results are given as odds ratio (OR) or hazard ratio and 95% confidence intervals

Day 90	mRS				Death			
	Unadjusted	*P*	Adjusted	*P*	Unadjusted	*P*	Adjusted	*P*
**Sodium** a	**0.46 (0.26, 0.81)**	**0.007**	**0.49 (0.28, 0.87)**	**0.015**	0.71 (0.30, 1.68)	0.43	0.81 (0.34, 1.93)	0.64
**Urea** a	**5.31 (2.36, 11.95)**	**<0.001**	**3.43 (1.42, 8.32)**	**0.006**	**3.30 (1.30, 8.38)**	**0.012**	**4.55 (1.51, 13.66)**	**0.007**
**eGFR** a	**0.89** **(0.81, 0.98)**	**0.021**	0.93 (0.83, 1.04)	0.23	0.90 (0.75, 1.08)	0.27	0.84 (0.68, 1.04)	0.11
**Urea : creatinine**	**1.07 (1.02, 1.11)**	**0.002**	1.03 (0.99, 1.08)	0.13	1.02 (0.97, 1.08)	0.45	1.01 (0.95, 1.08)	0.67
**Osmolarity A** a	0.90 (0.67, 1.20)	0.46	0.80 (0.60, 1.09)	0.16	1.21 (0.69, 2.12)	0.51	1.11 (0.65, 1.88)	0.70
**Osmolarity B** a	0.88 (0.66, 1.17)	0.38	0.80 (0.60, 1.08)	0.15	1.17 (0.66, 2.08)	0.59	1.10 (0.64, 1.88)	0.74
**Haematocrit** b	0.65 (0.41, 1.05)	0.08	0.74 (0.44, 1.25)	0.26	1.64 (0.79, 3.38)	0.18	1.42 (0.62, 3.26)	0.41
**Haemoglobin** a	0.89 (0.79, 1.01)	0.08	0.92 (0.80, 1.05)	0.21	1.13 (0.93, 1.37)	0.24	1.06 (0.85, 1.34)	0.60
**Red cell count**	0.94 (0.62, 1.44)	0.79	0.91 (0.58, 1.45)	0.70	1.79 (0.95, 3.36)	0.07	1.60 (0.81, 3.15)	0.18

Significant (*P* < 0.05) results in bold

eGFR, estimated glomerular filtration rate; GTN, glyceryl trinitrate

a OR per 10‐unit change

b OR per 0.1 l l^–1^ change

The rate of venous thromboembolism by day 7 in the population studied was low (*n* = 2), and therefore further analysis to establish any association with markers of dehydration was not deemed appropriate.

## Discussion

In the present preplanned substudy of patients with acute stroke, measures of dehydration ranged between 1.0% and 55.6%. There was no difference in BP change in dehydrated patients between those randomized to GTN *vs*. no GTN, and between those who continued *vs*. stopped their prestroke antihypertensive agents. Further, no differences in neurological status or clinical events at day 7 in dehydrated patients were seen across randomized groups. In a multivariable analysis, we found that increased urea was associated with an unfavourable shift in mRS and more death at day 90 overall. No consistent findings were noted for other markers of dehydration.

Conventional medical teaching suggests that giving antihypertensive medication, including nitrates, in the context of dehydration may lead to precipitous drops in BP. However, in this cohort of acute stroke patients, GTN did not have this effect. This incongruity may stem from the dose used (5 mg) and route of administration (transdermal), which is supported by the modest BP‐lowering effect seen across the trial in those randomized to GTN (7/3.5 mmHg lower after the first dose than those randomized to no GTN) 4. In addition, continuing prestroke antihypertensive agents did not cause large falls in BP in the context of dehydration, compared with stopping antihypertensive agents. The borderline interactions seen in relation to GTN and mRS and death at day 90 in those with raised urea are not supported by the neutral effects seen on haemodynamics across multiple dehydration markers. Such effects were not seen in those randomized to GTN with raised urea : creatinine and may, therefore, represent chance. More data are needed to confirm or refute this finding.

Several biochemical markers of dehydration were assessed using this dataset; only increasing serum urea was associated with 90‐day clinical outcome after acute stroke. In an earlier observational study of 2042 patients within 48 h of stroke, raised urea at baseline was associated with an increased risk of death up to 7 years later 20. In addition, raised creatinine and urea : creatinine ratio, and reduced creatinine clearance, at baseline were associated with higher mortality risk 20. Similarly, elevated urea on admission was associated with a higher mortality rate during initial hospitalization in a cohort of 388 stroke patients 21.

Other groups have noted associations between several markers of dehydration, other than urea, and outcome in acute stroke. First, an elevated blood urea nitrogen : creatinine ratio has been associated with poor neurological outcome and worse functional outcome in 2570 patients with acute ischaemic stroke 11. Similarly, in a UK cohort of 2591 acute stroke patients, a raised urea : creatinine ratio was associated with an increased likelihood of being dead or dependent at hospital discharge 10. Second, elevated plasma osmolality on admission has been associated with increased mortality after stroke 12. Third, reduced baseline eGFR has been associated with poor functional outcome after both ischaemic stroke 22 and intracerebral haemorrhage 23, and long‐term mortality and new cardiovascular morbidity over a 10‐year period in acute stroke overall 24. Last, urine specific gravity has been identified as a predictor of early neurological deterioration within 3 days of acute ischaemic stroke 25. In summary, although a number of dehydration markers have been associated with outcome after stroke, there are no consistent findings for any particular marker, perhaps reflecting that there is no gold standard measure of dehydration.

The strengths of the present study included the variety of blood dehydration parameters assessed, and the prespecified nature of this analysis within the context of a large randomized, controlled trial with almost complete follow‐up. Nevertheless, there were certain limitations. First, the data were limited to two sites and therefore might not be able to be extrapolated to larger datasets. Extrapolation to more severely dehydrated stroke patients may not be appropriate as patients with overt clinical dehydration may have been less likely to be recruited into ENOS. Further, the relatively small population studied meant that the analyses of clinical outcomes, although exploratory, were underpowered, and therefore the findings may represent chance. Second, although prognostic factors were adjusted for in analyses, we cannot exclude the possibility that the associations seen were due to chance or confounded by other factors such as infection, vomiting, medication and comorbidities. We did not collect information on fluid supplementation prior to or following blood being taken, which may have influenced the participant's fluid status, dehydration status and clinical outcomes measured. Further, diuretic use prestroke has been associated with dehydration in patients presenting with acute stroke 10, which may have confounded our results involving the assessment of GTN *vs*. no GTN. However, we did not see any significant associations between continuing prestroke antihypertensive agents and outcome in dehydrated stroke patients in this cohort. Third, no adjustment was made for multiplicity of testing, and therefore some findings may represent chance, highlighted by the opposite directions of association with day 90 outcomes seen for sodium and urea, and neutral findings in relation to the creatinine and urea : creatinine ratio. Last, whether the negative effects of dehydration in acute stroke patients are seen in the longer term was not answered by the present analysis of outcomes assessed at a relatively early stage poststroke.

In summary, transdermal GTN or continuation of pre‐existing antihypertensive treatment did not cause precipitous drops in BP in dehydrated acute stroke patients. This is of reassurance and supports the use of GTN in acute stroke prior to blood markers of dehydration being available. The ongoing Rapid Intervention with Glyceryl trinitrate in Hypertensive stroke Trial‐2 (RIGHT‐2) is assessing the safety and efficacy of GTN in the ambulance within 4 h of symptom onset and will add to these data in the ultra‐acute setting 26. Dehydration, when measured as urea, was associated with poor clinical outcomes after acute stroke. Whether rehydration of dehydrated acute stroke patients has the potential to improve clinical outcomes requires further assessment in randomized controlled trials.

## Competing Interests

There are no competing interests to declare.

*We thank the participants and investigators who took part in the ENOS trial. This work was supported by the Medical Research Council [grant number G0501797]. J.P.A. is funded by NIHR TARDIS (10/104/24) and BHF RIGHT‐2 (CS/14/4/30972). P.M.B. is Chief Investigator of MRC ENOS, Stroke Association Professor of Stroke Medicine, and is a NIHR Senior Investigator.*

## Contributors

C.K.B. performed data collection. J.P.A. performed the analyses and wrote the first draft of the manuscript. P.M.B. conceived the analyses and is guarantor of the study. All authors commented upon and approved the final manuscript.

## Supporting information


**Table S1** Effects of glyceryl trinitrate (GTN) *vs*. non‐GTN, and effects of continue *vs*. stop antihypertensive treatment on change in neurological status and clinical events during the first 7 days and outcome at 3 months, by hydration status, given by urea : creatinine ratio. Multiple linear regression, binary logistic regression, ordinal logistic regression or Cox proportional hazards regression with adjustment for age, sex and time to randomization. Data are given as *n* (%), mean (standard deviation), mean difference (MD), odds ratio (OR) or hazard ratio (HR) with 95 confidence intervals (CIs)
**Table S2** Unadjusted and adjusted relationships between modified Rankin Scale (mRS) or death, and baseline markers of dehydration (in addition to those presented in Table 4). Analysis by ordinal logistic regression or Cox proportional hazards regression; with adjustment for age, sex, systolic blood pressure, stroke severity (Scandinavian Stroke Scale), time from onset to randomization, continue/stop and GTN/no GTN. Results are odds ratio (OR) or hazard ratio (HR) and 95% confidence intervals (CIs). Significant (*P* < 0.05) results in bold
