# Supplementary material for: Impact of hydration status on haemodynamics, effects of acute blood pressure‐lowering treatment, and prognosis after stroke
Source: Br J Clin Pharmacol. 2018 Oct 10;84(12):2914–22. doi: 10.1111/bcp.13761 (PMC6256053; doi:10.1111/bcp.13761)
Supplement: Supplementary file 1 — Table S1 Effects of glyceryl trinitrate (GTN) vs. non‐GTN, and effects of continue vs. stop antihypertensive treatment on change in neurological status and clinical events during the first 7 days and outcome at 3 months, by hydration status, given by urea : creatinine ratio. Multiple linear regression, binary logistic regression, ordinal logistic regression or Cox proportional hazards regression with adjustment for age, sex and time to randomization. Data are given as n (%), mean (standard deviation), mean difference (MD), odds ratio (OR) or hazard ratio (HR) with 95 confidence intervals (CIs) Table S2 Unadjusted and adjusted relationships between modified Rankin Scale (mRS) or death, and baseline markers of dehydration (in addition to those presented in Table 4). Analysis by ordinal logistic regression or Cox proportional hazards regression; with adjustment for age, sex, systolic blood pressure, stroke severity (Scandinavian Stroke Scale), time from onset to randomization, continue/stop and GTN/no GTN. Results are odds ratio (OR) or hazard ratio (HR) and 95% confidence intervals (CIs). Significant (P < 0.05) results in bold [file BCP-84-2914-s001.docx]

**Impact of hydration status on haemodynamics, effects of acute blood pressure lowering treatment, and prognosis after stroke supplementary tables**

**Supplementary table 1.** Effects of GTN vs. non-GTN, and effects of Continue v. Stop antihypertensive treatment on change in neurological status and clinical events during the first 7 days and outcome at 3 months, by hydration status, given by urea:creatinine ratio. Multiple linear regression, binary logistic regression, ordinal logistic regression or Cox proportional hazards regression with adjustment for age, sex and time to randomisation. Data are n (%), mean (SD), mean difference (MD), odds ratio (OR) or hazard ratio (HR) with 95 confidence intervals (CI).

|  | **Ur:Cr** | **All** | **GTN** | **No GTN** | **OR/MD/HR  (95% CI)** | **p** | **p _interaction_** | **Cont.** | **Stop** | **HR/OR/MD/HR (95% CI)** | **p** | **p _interaction_** |
| --- | --- | --- | --- | --- | --- | --- | --- | --- | --- | --- | --- | --- |
| Day 7 |  | n=297 | n=154 | n=143 |  |  |  | n=70 | n=72 |  |  |  |
| ∆SSS 0-7 | >20 | 3.3 (10.9) | 2.8 (11.7) | 3.8 (10.3) | -0.77 (-6.50, 4.96) | 0.79 | 0.95 | 3.7 (10.3) | 2.4 (12.9) | 1.30 (-7.84, 10.44) | 0.78 | 0.54 |
|  | <20 | 5.3 (10.0) | 5.2 (10.6) | 5.5 (9.3) | -0.87 (-3.67, 1.94) | 0.54 | - | 2.8 (11.8) | 3.7 (10.5) | -0.80 (-5.51, 3.91) | 0.74 | - |
| Hypotension   (%) | >20 | 3 (4.0) | 2 (5.6) | 1 (2.6) | 2.37 (0.18, 30.99) | 0.51 | - | 2 (7.7) | 1 (5.3) | 1.46 (0.10, 20.81) | 0.78 | 0.65 |
|  | <20 | 6 (2.7) | 6 (5.1) | 0 (0) | - | - | - | 2 (4.5) | 3 (5.7) | 1.07 (0.15, 7.70) | 0.95 | - |
| Hypertension   (%) | >20 | 3 (4.0) | 2 (5.6) | 1 (2.6) | 2.92 (0.23, 37.98) | 0.41 | 0.63 | 0 (0) | 1 (5.3) | - | - | - |
|  | <20 | 10 (4.5) | 6 (5.1) | 4 (3.8) | 1.40 (0.37, 5.30) | 0.62 | - | 2 (4.5) | 2 (3.8) | 1.38 (0.16, 11.58) | 0.77 | - |
| Headache (%) | >20 | 10 (13.3) | 8 (22.2) | 2 (5.1) | 7.38 (1.28, 42.67) | 0.026 | 0.16 | 3 (11.5) | 2 (10.5) | 0.92 (0.11, 7.85) | 0.94 | 0.84 |
|  | <20 | 42 (18.9) | 27 (22.9) | 15 (14.4) | 1.74 (0.86, 3.52) | 0.13 | - | 4 (9.1) | 5 (9.4) | 1.07 (0.26, 1.38) | 0.93 | - |
| Day 90 |  |  |  |  |  |  |  |  |  |  |  |  |
| Functional   outcome   (mRS)* | >20 | 3.6 (1.7) | 3.7 (1.8) | 3.5 (1.5) | 1.03 (0.46, 2.33) | 0.94 | 0.84 | 3.5 (1.6) | 3.6 (1.8) | 0.73 (0.25, 2.19) | 0.58 | 0.99 |
|  | <20 | 3.0 (1.7) | 3.0 (1.7) | 2.9 (1.7) | 1.23 (0.77, 1.97) | 0.38 | - | 3.2 (1.8) | 3.3 (1.6) | 0.88 (0.43, 1.77) | 0.71 | - |
| Death (%) | >20 | 13 (17.3) | 8 (22.2) | 5 (12.8) | 2.66 (0.85, 8.34) | 0.09 | 0.53 | 3 (11.5) | 4 (21.1) | 0.57 (0.12, 2.68) | 0.48 | 0.44 |
|  | <20 | 21 (9.5) | 13 (11.1) | 8 (7.7) | 1.52 (0.62, 3.68) | 0.36 | - | 6 (13.6) | 7 (13.2) | 1.15 (0.36, 3.63) | 0.81 | - |

* ordinal logistic regression. BP: blood pressure; CI: confidence interval; GTN: glyceryl trinitrate; mRS: modified Rankin Scale; OR: odds ratio; SD: standard deviation; SSS: Scandinavian Stroke Scale.

Ur:Cr= Urea: creatinine ratio high >20, normal <20

**Supplementary table 2**. Unadjusted and adjusted relationships between modified Rankin Scale (mRS) or death and baseline markers of dehydration (in addition to table 4). Analysis by ordinal logistic regression or Cox proportional hazards regression; with adjustment for age, sex, systolic blood pressure, stroke severity (Scandinavian Stroke Scale), time from onset to randomisation, continue/stop and GTN/no GTN. Results are odds ratio (OR) or hazard ratio (HR) and 95% confidence intervals (CI). Significant (p<0.05) results in bold.

| Day 90 | mRS | | | | Death | | | |
| --- | --- | --- | --- | --- | --- | --- | --- | --- |
|  | Unadjusted | p | Adjusted | p | Unadjusted | p | Adjusted | p |
| Sodium >145 | 1.21  (0.16, 8.94) | 0.85 | 0.60  (0.08, 4.68) | 0.63 | - | - | - | - |
| Potassium | 0.87  (0.55, 1.36) | 0.54 | 1.34  (0.84, 2.17) | 0.22 | 1.18  (0.55, 2.55) | 0.67 | 1.45  (0.70, 2.99) | 0.32 |
| Urea >7.5 | **1.71**  **(1.06, 2.76)** | **0.028** | 1.47  (0.88, 2.44) | 0.14 | 1.67  (0.85, 3.29) | 0.14 | 1.95  (0.94, 4.03) | 0.07 |
| Urea tertiles |  |  |  |  |  |  |  |  |
| <5.4 | 1.00 | - | 1.00 | - | 1.00 | - | 1.00 | - |
| 5.4-6.9 | 1.48  (0.90, 2.42) | 0.12 | 1.11  (0.67, 1.86) | 0.84 | 1.15  (0.42, 3.17) | 0.79 | 1.42  (0.49, 4.11) | 0.52 |
| >6.9 | **2.04**  **(1.25, 3.34)** | **0.004** | 1.69  (0.99, 2.89) | 0.056 | 1.62  (0.64, 4.07) | 0.31 | 1.76  (0.64, 4.81) | 0.27 |
| Creatinine* | 1.04 (0.98, 1.11) | 0.18 | 1.06 (0.98, 1.13) | 0.13 | 1.08 (0.99, 1.17) | 0.07 | **1.11 (1.02, 1.20)** | **0.019** |
| eGFR <30 | 2.71  (0.47, 15.55) | 0.26 | 6.54  (0.97, 44.04) | 0.053 | 1.97  (0.27, 14.40) | 0.51 | 2.44  (0.30, 20.02) | 0.41 |
| eGFR |  |  |  |  |  |  |  |  |
| <60.3 | 1.48  (0.91, 2.43) | 0.12 | 1.19  (0.67, 2.10) | 0.55 | 1.13  (0.50, 2.53) | 0.77 | 1.49  (0.56, 3.97) | 0.43 |
| 60.3-77.8 | 1.53  (0.94, 2.51) | 0.09 | 1.20  (0.71, 2.01) | 0.49 | 0.69  (0.27, 1.80) | 0.45 | 0.60  (0.22, 1.64) | 0.60 |
| >77.8 | 1.00 | - | 1.00 | - | 1.00 |  | 1.00 |  |
| Glucose* | **4.27 (1.27, 14.32)** | **0.019** | 1.61  (0.46, 5.60) | 0.45 | 3.74  (0.74, 18.90) | 0.11 | 1.58  (0.22, 11.15) | 0.65 |
| Haematocrit, increased | 1.45  (0.35, 6.04) | 0.61 | 0.74  (0.17, 3.23) | 0.69 | 1.17  (0.16, 8.54) | 0.88 | 0.82  (0.10, 6.39) | 0.85 |
| Haematocrit |  |  |  |  |  |  |  |  |
| <0.4 | 1.00 | - | 1.00 | - | 1.00 | - | 1.00 | - |
| 0.4-0.44 | 0.80  (0.49, 1.31) | 0.38 | 0.97  (0.58, 1.6) | 0.90 | 0.89  (0.38, 2.12) | 0.80 | 1.10  (0.44, 2.75) | 0.83 |
| >0.44 | 0.67  (0.41, 1.1) | 0.11 | 0.76  (0.45, 1.31) | 0.33 | 2.15  (0.98, 4.71) | 0.057 | 2.13  (0.87, 5.21) | 0.10 |

*OR per 10 units change. eGFR: estimated glomerular filtration rate; GTN: glyceryl trinitrate.
